# Inhibitory Effect of Osthole from *Cnidium monnieri* (L.) *Cusson* on *Fusarium oxysporum,* a Common Fungal Pathogen of Potato

**DOI:** 10.3390/molecules26133818

**Published:** 2021-06-23

**Authors:** Hongli Zheng, Yahan Chen, Qiuli Guo, Hong Wei, Jianying Yue, Hongyou Zhou, Mingmin Zhao

**Affiliations:** 1 College of Horticulture and Plant Protection, Inner Mongolia Agricultural University, Hohhot 010018, China; Zhlfcy66@126.com (H.Z.); 18447053473@163.com (Q.G.); weihongwl@163.com (H.W.); yuejianying2018@163.com (J.Y.); 2 College of Plant Protection, Gansu Agricultural University, Lanzhou 730070, China; yhchen1018@nwafu.edu.cn

**Keywords:** *Cnidium monnieri*, osthole, *Fusarium oxysporum*, potato

## Abstract

*Fusarium* wilt of potato is one of the most common diseases of potato in China, and is becoming a serious threat in potato production. It has been reported that osthole from *Cnidium monnieri* (*L.*) *Cusson* can inhibit plant pathogens. Here, we test the anti-fungal activity of *C. monnieri* osthole against *Fusarium oxysporum* in potatoes. The results showed that at a concentration of 5 mg/mL, osthole was able to obviously inhibit mycelial growth of *F. oxysporum*. We found that osthole caused changes of mycelial morphology, notably hyphal swelling and darkening. Osthole significantly reduced the spore germination of *Fusarium* by 57.40%. In addition, osthole also inhibited the growth of other pathogens such as *Fusarium moniliforme* J. Sheld, *Thanatephorus cucumeris* Donk, and *Alternaria alternata* (Fr.) Keissl, but not *Alternaria solani* Jonesetgrout and *Valsa mali* Miyabe and G. Yamada. Our results suggest that osthole has considerable potential as an agent for the prevention and treatment of potato *Fusarium* wilt.

## 1. Introduction

Potato Fusarium wilt is a typical soil-borne disease that leads to severe wilt symptoms, ultimately resulting in plant death, which directly affects the yield and quality of potatoes [1,2,3]. This disease is widely distributed worldwide and generally causes about 30% reduction in production. According to the reports of Rakhimov et al. (2000), potato *Fusarium* wilt is mainly caused by either of five different *Fusarium* varieties, i.e., *Fusarium oxysporum* Schlecht, *F. solani* (Mart.) Sacc., *F. moniliforme* Sheld, *F. sambucinum* Fuckel, and *F. nivale* (Fr.) Ces. *F. oxysporum* Schlecht, *F. solani* (Mart.) Sacc., and *F. nivale* (Fr.) Ces are the agents of potato *Fusarium* wilt in Inner Mongolia, China.

Because the disease is a soil-borne disease and the pathogen exhibits a strong resistance to stress, it is difficult to treat soil with chemicals in the field [4]. At present, the control of potato *Fusarium* wilt mainly relies on agricultural and chemical seed dressing methods. Environmental pollution and the persistence of fungicide residues in potato tubers are important concerns in the control of potato *Fusarium* wilt. Thus, plant-derived fungicides are promising alternatives because they are largely non-phytotoxic, easily biodegradable, and environmentally safe [5]. Plant compounds with antifungal properties include proteins, alkaloids, flavonoids, phenols, essential oils, and polysaccharides [5,6,7,8,9,10]. *Cnidium monnieri* (*L.*) *Cusson* is a traditional Chinese medicinal plant widely distributed throughout China [11,12,13]. A total of 429 chemical compounds have been detected in *C. monnieri*, and of these 56 have been chemically identified [14]. Osthole is an *O*-methylated coumarin isolated and purified from the seeds of *C. monnieri*. It was widely shown to have pharmacological functions, such as anti-allergic, anti-pruritic, anti-bacterial, anti-dermatophytic, anti-osteoporotic, and anti-fungal activities in humans [15,16,17,18,19]. Furthermore, osthole was found to enhance osteogenesis in osteoblasts by elevating transcription factor osterix via cyclic adenosine monophosphate (cAMP)/the cAMP response element-binding protein (CREB) signaling [20].

However, its anti-fungal activity against plant pathogens remains unknown. Previously, we identified the osthole from *C. monnieri* and found that osthole inhibited the infection of *Nicotiana glutinosa* by the Tobacco mosaic virus (TMV) [21]. This led us to wonder whether osthole could inhibit the pathogen of potato *Fusarium* wilt. In this study, we investigated the effects of osthole on mycelium growth and spore production of *Fusarium oxysporum* Schlecht. The activity of osthole against other fungal pathogens was also tested with the aim of exploring possible applications of osthole in the control of plant fungal diseases.

## 2. Results

### 2.1. Osthole Effects on the Growth of F. oxysporum

**Figure 1:**
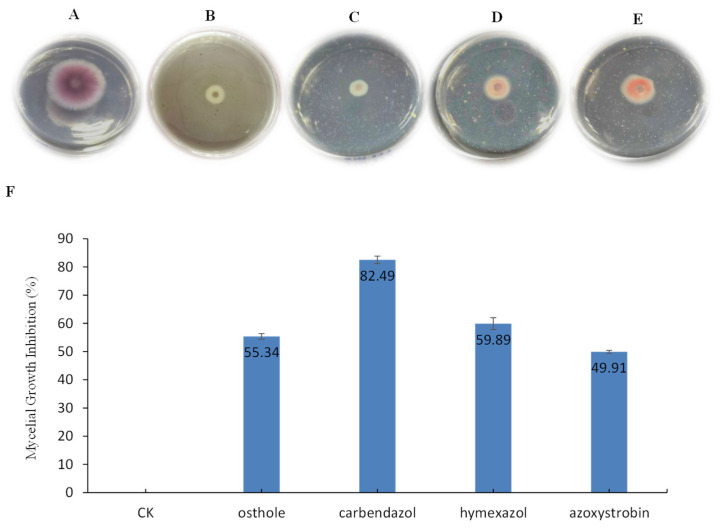
The inhibitory effect of osthole on *Fusarium oxysporum* at 3 days after inoculation. (**A**) CK, potato dextrose agar (PDA) medium; (**B**) PDA medium plus 5 mg/mL osthole; (**C**) PDA medium plus 5 mg/mL carbendazol wettable powder; (**D**) PDA medium plus 5 mg/mL hymexazol aqueous solution; (**E**) PDA medium plus 5 mg/mL azoxystrobin; (**F**) The mycelial growth inhibition are shown. CK, PDA medium. The bars on the columns indicate standard deviation.

The effect of osthole on *F. oxysporum* was examined on potato dextrose agar (PDA) plates containing 5 mg/mL osthole. Compared with the control (water), the mycelial growth was significantly inhibited in plates containing osthole, as evidenced by mycelial growth inhibition at 3 days after inoculation (Figure 1A). The mycelial growth inhibition was 55.34%, a value that was comparable to that observed with three other compounds of common use as fungal inhibitors, carbendazol wettable powder (82.49%), hymexazol aqueous solution (59.89%), and azoxystrobin (49.91%) (Figure 1B). This indicates that osthole is able to efficiently inhibit the mycelial growth of *Fusarium oxysporum*.

### 2.2. The Dose-Dependent Inhibitory Activity of Osthole on the Growth of F. oxysporum

**Figure 2:**
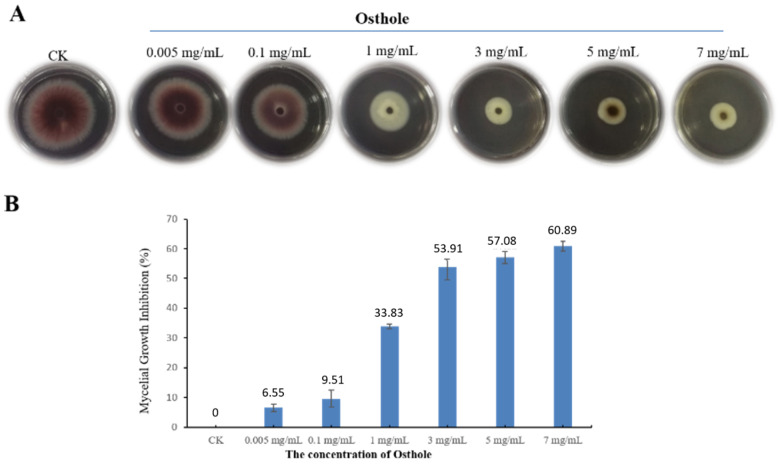
The inhibitory effect of osthole at different concentrations on *F. oxysporum*. The colony morphology (**A**) and the mycelial growth inhibition (**B**) of *F. oxysporum* in media containing osthole, 5 days after inoculation.

To determine the optimal concentration of osthole for inhibiting mycelial growth, we diluted osthole in PDA medium (resulting in solutions of various concentrations) and inoculated the *F. oxysporum* mycelia. We found that the mycelial growth was considerably reduced when the concentration of osthole was increased to 1 mg/mL (Figure 2A,B). The mycelial growth inhibition in plates containing 0.005 mg/mL (6.55%) and 0.1 mg/mL (9.51%) osthole were no significant difference to those in the control. When the concentration of osthole reached 1 mg/mL, themycelial growth inhibition sharply increase ed to 33.83%. The inhibitory effect of osthole on the growth of *F. oxysporum* mycelia was more prominent at concentrations of 3 mg/mL or more (5 mg/mL and 7 mg/mL). Moreover, the mycelia morphology also exhibited pronounced malformations.

Thus, a significant positive correlation was observed between the concentration of osthole and its inhibitory effects on the growth of *F. oxysporum* mycelia.

### 2.3. Effects of Osthole on Mycelia Morphology and Spore Germination of F. oxysporum

**Figure 3:**
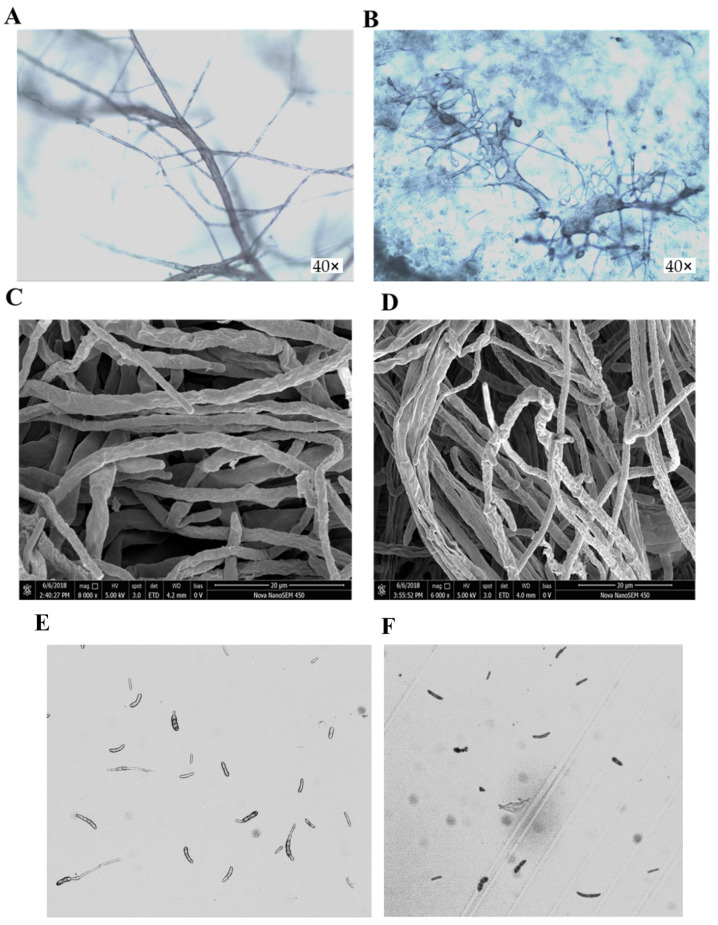
Effect of osthole on the morphology of *F. oxysporum* mycelia and *spore* germination. (**A**): CK, images of *Fusarium oxysporum* mycelia on PDA medium acquired using optical microscope; (**B**): images of *Fusarium oxysporum* mycelia on PDA medium plus 5 mg/mL osthole acquired using optical microscope. Mycelial morphology was observed by scanning electron microscopy and is shown in (**C**) (CK) and (**D**) (5 mg/mL osthole). (**E**): CK, *Spore* germination of *Fusarium oxysporum* on PDA liquid medium; (**F**): *Spore* germination of *Fusarium oxysporum* on PDA liquid medium plus 3 mg/mL osthole.

Mycelia morphology and spore germination of *F. oxysporum* were observed by optical microscopy and scanning electron microscopy (SEM). We found that osthole (5 mg/mL) induced morphological changes in *F. oxysporum*, characterized by shrunken hyphae with abnormal shape, vesicles, distortion, or empty cells devoid of cytoplasm in the mycelia (Figure 3B,D). Osthole induced a visible increase in mycelial branching, enlargement of apical branches, and malformation in *F. oxysporum*. No swelling or distortion was observed in the untreated controls (Figure 3A,C). Osthole also significantly inhibited the germination of *F. oxysporum* spores (Figure 3E,F). After incubation at 30 °C for 18 h, the germination of *F. oxysporum* spores was 14.45% in PDA medium containing 5 mg/mL osthole, versus 33.92% in the control (Table 1). This suggests that osthole affects mycelial growth and spore germination.

### 2.4. Inhibitory Activity of Osthole against Other Fungal Pathogens

**Figure 4:**
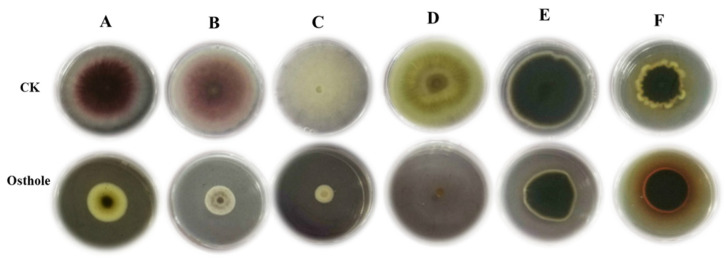
Inhibitory effect of osthole on several fungal pathogens. (**A**) *F. oxysporum* Schlecht; (**B**) *F. moniliforme* Sheld); (**C**) *T. cucumeris* Donk; (**D**) *V. mali* Miyabeet Yamada); (**E**) *A. solani* Jonesetgrout; (**F**) *A. alternata* Keissler. PDA containing osthole (5 mg/mL) was used. CK, PDA dishes were added with the same volume of Tween-20 and distilled water (1:1000 *v/v*).

In order to extend the possibility of osthole application in the control of other fungal diseases in plant, the inhibitory activity of osthole was tested against six fungal pathogens using the plate confrontation test. Osthole (5 mg/mL) could effectively inhibit the growth of *F. oxysporum* Schlecht, *F. moniliforme* Sheld, *Thanatephorus cucumeris* Donk, and *F. Valsa* mali Miyabeet Yamada, but not of *A. alternata* Keissler and *Alternaria solani* Jonesetgrout (Figure 4). The inhibition rate of *F. oxysporum* Schlecht, *F. moniliforme* Sheld, *T. cucumeris* Donk, and *V. mali* Miyabeet Yamada were 55.34%, 70.66%, 75.90%, and 90.36%, respectively, whereas no significant inhibitory activity was observed against *A. alternata* Keissler (37.60%) and *A. solani* Jonesetgrout (37.05%) (Table 2).

## 3. Materials and Methods

### 3.1. Test Fungal Pathogens and Storage

*F. oxysporum* Schlecht, *F. moniliforme* Sheld, *T. cucumeris* Donk, *V. mali* Miyabeet Yamada, *A. alternata* Keissler, and *A. solani* Jonesetgrout were provided by the Microbiology Institute of Shaanxi, Shaanxi Province, China. *V. mali* Miyabe and G. Yamada was provided by the State Key Laboratory of Crop Stress Biology for Arid Areas, Northwest A and F University, Yangling, Shanxi, China. *F. oxysporum* Schlecht was obtained from the Laboratory of Phytopathology, Inner Mongolia Agricultural University, Huhhot Inner Mongolia, China. The fungi were stored at 4 °C on PDA (200 g potato peelings, 20 g dextrose, 15 g agar, and 1000 mL distilled water, pH 7.0) and grown in culture medium at 25 °C.

### 3.2. Chemicals and Materials

The osthole was extracted from *C. monnieri* (100 g) with 500 mL 90% methanol as described by Chen et al. 2019 [21].

Carbendazol WP (50%) was obtained from Jiangsu Sanshan Pesticide Co., Ltd. (Jiangsu, China). Hymexazol aqueous (30%) was purchased from Heyi chemical Co. LTD (Jiangxi, China). Azoxystrobin (25%) was purchased from Yinuo biochemical Co. LTD (Hebei, China). We chose these chemicals because they are effective antifungal reagents commonly used in controlling *F. oxysporum* in potato in China [22,23].

### 3.3. Inhibitory Activity of Osthole on the Growth of F. oxysporum Schlecht

We tested the effects of osthole on the growth of *F. oxysporum* Schlecht mycelia in PDA medium cultures using the agar dilution method [5]; control PDA plates without osthole were inoculated with equivalent fungi. First, osthole was dissolved in Dimethyl sulfoxide (DMSO) (1000 mg/mL) and diluted to required concentration with Tween-20 and distilled water (1:1000 *v/v*). We prepared the PDA plate containing 5 mg/mL of osthole. As the control, PDA dishes were added with the same volume of Tween-20 and distilled water (1:1000 *v/v*). The *F. oxysporum* Schlecht was inoculated on center of the plate. Three days later, the diameters of colonies were measured and the growth inhibition rates were calculated [5].

A series dilutions of osthole (0.005, 0.01, 0.10, 1.00, 3.00, 5.00, and 7.00 mg/mL) was prepared as described by Chen et al. in 2019 [5]. A 6 mm diameter disc of *F. oxysporum* Schlecht was inoculated at the center of the PDA plate. After incubation for 7 days at 25 ± 2 °C, the diameter of the inhibition zone was measured. Twelve plates of each group were tested. Each treatment was performed three times. We used the formula proposed by Chen et al. in 2019 to calculate mycelial growth inhibition [5].

### 3.4. Mycelia Observation by Scanning Electron Microscopy (SEM)

*F. oxysporum* Schlecht growing on PDA containing osthole (5 mg/mL) for 7 days were observed by SEM and their morphology was compared with that of the controls. Samples were prepared using the protocol proposed by Chen et al. (2019). All mycelia samples were observed using a Nova NanoSEM 450 (FEI Co. LTD, Hillsboro, OR, USA) at 5.00 kV of magnification.

### 3.5. Effects of Osthole on the Germination of F. oxysporum Schlecht Spores

Pathogen spore suspension at a concentration of ~40 spore/view (low-power scan; 10 × 10) and osthole at the 5 mg/mL concentration were mixed in equal volumes. One droplet was placed on a microscope slide coated with sterile collodion. Each treatment was repeated three times. The spores were considered as germinated when the length of germ tube exceeded the spore radius [24,25].

### 3.6. Statistical Analysis

All data are presented as mean values plus/minus SD from three independent replicates. The data were analyzed using a Data Processing System 15.10 (Hefei, China). The significance of the differences between the four means was determined using Duncan’s new complex range method at the 5% level.

## 4. Discussion

Osthole is a natural coumarin present in the fruits of *Cnidium monnieri* (L.) Cusson [26]. Crude extracts from the dried fruits of *C. monnieri* have been extensively used as a traditional Chinese medicine to treat various conditions such as osteoporosis [27], pulmonary inflammation [28] and certain skin diseases [15,18]. Thus, osthole is an important constituent of the dried fruits and has been recognized as a promising compound in drug discovery research. In particular, we previously identified the osthole from *C. monnieri* and and reported an obvious inhibition effect on TMV infection [21]. This stimulated us to test whether osthole could have antifungal activity in plant diseases, especially those soil-borne diseases.

In this study, we have chosen *F. oxysporum* Schlecht to test the antifungal effect of osthole. The results showed that at a concentration of 1 mg/mL, osthole was able to inhibit mycelial growth of *F. oxysporum* according to the increased growth inhibition. With the concentration increasing from 1 mg/mL to 7 mg/mL, no obvious difference of inhibition effect was observed. Based on this, 5 mg/mL of osthole was used for most assays. The changes of mycelia morphology also support an antifungal effect of osthole. We also performed the assay to explore the possibility whether osthole could affect the spore germination of *Fusarium*. The inhibition rate of 57.40% was obtained. These results are consistent with former reports of morphological alterations of fungal hyphae after treatment with chemical fungicides, chitosan, and natural botanicals [22,29,30,31]. These data greatly strengthen the possibility of utilizing osthole as plant-derived reagents in antifungal control in potato fusarium disease.

To further practical application points, we also explored the inhibition effect of osthole on other fungal pathogens in PDA plates. The results showed that osthole also inhibited the growth of other pathogens such as *Fusarium moniliforme* J. Sheld, *Thanatephorus cucumeris* Donk, and *Alternaria alternata* (Fr.) Keissl, but not the growth of *Alternaria solani* Jonesetgrout and *Valsa mali* Miyabe and G. Yamada. This suggested that osthole has a potential application in the prevention and treatment of some plant fungal diseases, but not all.

In conclusion, even though we found osthole could potentially be used as an inhibitory agent against plant fungal pathogens, its antifungal mechanism and crucial application points should be further studied before it can be used reliably.

## Figures and Tables

**Table 1:** Germination of *F. oxysporum* spores.

Treatment	Total Spore Number	Germinated Spore Number	Germination Rate (%)	Inhibition Rate (%)
CK	39.80 ± 2.64	13.5 ± 1.95	33.92 ± 2.05	
osthole	34.60 ± 4.51	5 ± 3.06	14.45 ± 4.27	57.40 ± 3.75

Values are presented as the mean ± SD.

**Table 2:** Mycelial diameter of six pathogens in response to osthole treatment in vitro.

Pathogen	Mycelial Growth Inhibition Rate (%)
*F. oxysporum* Schlecht	55.34 ± 4.32
*F. moniliforme* Sheld	70.66 ± 1.47 *
*T. cucumeris* Donk	75.90 ± 0.73
*F. Valsa* mali Miyabeet Yamada	90.36 ± 2.88
*A. alternata* Keissler	37.60 ± 4.05 *
*A. solani* Jonesetgrout	37.05 ± 0.97

Values are presented as the mean ± SD. * indicates significant difference at *p* < 0.05 using Duncan’s new multiple range test. PDA containing osthole (5 mg/mL) was used.

## Data Availability

The study did not applicable.

## Sample Availability

Samples of the compounds are not available from the authors.

## References

[1] Rakhimov U.K., Khakimov A.K. (2000). Wilt of potatoes in Uzbekistan. Zashchita I Karantin Rastenii.

[2] Peng X.W., Zhu J.H. (2008). Species and Distribution of potato Fungal Diseases in Hebei Province, China. Chin. Potato J..

[3] An X.M., Hu J., Wu J.H., Liu Z.H., Meng M.L. (2017). Overview of pathogen causing potato Fusarium Wilt. Chin. Potato J..

[4] Khedher S.B., Mejdoub-Trabelsi B., Tounsi S. (2020). Biological potential of Bacillus subtilis V26 for the control of Fusarium wilt and tuber dry rot on potato caused by Fusarium species and the promotion of plant growth. Biol. Control.

[5] Chen Y.H., Lu M.H., Guo D.S., Zhai Y.Y., Miao D., Yue J.Y., Yuan C.H., Zhao M.M., An D.R. (2019). Antifungal Effect of Magnolol and Honokiol from Magnolia officinalis on *Alternaria alternata* Causing Tobacco Brown Spot. Molecules.

[6] Mi Y.Y., Byeong J.C., Jin C.K. (2013). Recent Trends in Studies on Botanical Fungicides in Agriculture. Plant Pathol..

[7] Kijjoa A., Pinto M.M.M., Tantisewie B., Herz W. (1989). A biphenyl type neolignan and biphenyl ether from Magnolia henryi. Phytochemistry.

[8] Du C.M., Wu Y.H., Zhao X.X., Zhu C.Y., Jiang G., Yan X.M. (2004). Recent development in research of natural antiphytoviral substances. Acta Tabacaria Sin..

[9] Chen Y.H., Ru B.L., Zhai Y.Y., Li J., Cheng J.L. (2018). Screening and inhibitory effects of plant extracts against Tobacco mosaic virus (TMV). J. Plant Prot..

[10] Zhao L., Feng C., Wu K., Chen W.B., Chen Y.J., Hao X.A., Wu Y.F. (2016). Advances and prospects in biogenic substances against plant virus: A review. Pestic. Biochem. Phys..

[11] Kitajima J., Aoki Y., Ishikawa T., Tanaka Y. (1999). Monoterpenoid glucosides of Cnidium monnieri fruit. Chem. Pharm. Bull..

[12] Oh H., Kim J.S., Song E.K., Cho H., Kim D.H., Park S.E., Lee H.S., Kim Y.C. (2002). Sesquiterpenes with hepatoprotective activity from Cnidium monnieri on tacrine-induced cytotoxicity in Hep G2 cells. Planta Med..

[13] Zhao J.Y., Zhou M., Liu Y., Zhang G.L., Luo Y.G. (2011). Chromones and coumarins from the dried fructus of Cnidium monnieri. Fitoterapia.

[14] Sun Y., Yang A., Lenon G.B. (2020). Phytochemistry, Ethnopharmacology, Pharmacokinetics and Toxicology of *Cnidium monnieri* (L.) Cusson. Int. J. Mol. Sci..

[15] Basnet P., Yasuda I., Kumagai N., Tohda C., Nojima H., Kuraishi Y., Komatsu K. (2001). Inhibition of itch-scratch response by fruits of Cnidium monnieri in mice. Biol. Pharm. Bull..

[16] Bao J.J., Xie M.L., Zhu L.J. (2011). Treatment of osthol on osteoporosis in ovariectomized rats. Chin. Pharm. Bull..

[17] Matsuda H., Ido Y., Hirata A., Ino Y., Naruto S., Amamiya T., Kubo M. (2002). Antipruritic effect of Cnidii monnieri Fructus (fruits of Cnidium monnieri Cusson). Biol. Pharm. Bull..

[18] Matsuda H., Tomohiro N., Ido Y., Kubo M. (2002). Anti-allergic effects of Cnidii monnieri fructus (dried fruits of Cnidium monnieri) and its major component, osthol. Biol. Pharm. Bull..

[19] Li Y.M., Jia M., Li H.Q., Zhang N.D., Wen X., Rahman K., Zhang Q.Y., Qin L.P. (2015). Cnidium monnieri: A Review of Traditional Uses, Phytochemical and Ethnopharmacological Properties. Am. J. Chin. Med..

[20] Zhang Z.R., Leung W.N., Li G., Kong S.K., Lu X., Wong Y.M., Chan C.W. (2017). Osthole Enhances Osteogenesis in Osteoblasts by Elevating Transcription Factor Osterix via cAMP/CREB Signaling In Vitro and In Vivo. Nutrients.

[21] Chen Y.H., Guo D.S., Lu M.H., Yue J.Y., Liu Y., Shang C.M., An D.R., Zhao M.M. (2019). Inhibitory Effect of Osthole from *Cnidium monnieri* on *Tobacco Mosaic Virus* (TMV) Infection in *Nicotiana glutinosa*. Molecules.

[22] Chen C., Chen Y., Wang C., Wang C., Zhang S., Zhang F. (2014). Fungicide selection in controlling potato blight. Guizhou Agric. Sci..

[23] Xue Y., Meng M., Hu J., Zhang X., Wang X. (2012). Determination for Toxicity of Six Fungicides to Fusarium Wilt Pathogen of Potato in vitro. Chin. Potato J..

[24] Sun P.P., Jia X.H., Cui J.C., Tong W., Wang W.H. (2016). Selection, Identification and Characterization of Actinomyces L-30 for theBiocontrol of Pear Gray Mold. Acta Hortic. Sin..

[25] Shan H.Y., Zhao M.M., Chen D.X., Chen J.L., Li J., Feng Z.Z., Ma Z.Y., An D.R. (2013). Biocontrol of rice blast by the phenaminomethylacetic acid producer of Bacillus methylotrophicus strain BC79. Crop Prot..

[26] Callahan B.N., Kammala A.K., Syed M., Yang C., Occhiuto C.J., Nellutla R., Chumanevich A.P., Oskeritzian C.A., Das R., Subramanian H. (2020). Osthole, a Natural Plant Derivative Inhibits MRGPRX2 Induced Mast Cell Responses. Front. Immunol..

[27] Zhang Q., Qin L., He W., Van Puyvelde L., Maes D., Adams A., Zheng H., De Kimpe N. (2007). Coumarins from *Cnidium monnieri* and their antiosteoporotic activity. Planta Medica.

[28] Kwak H.G., Lim H.B. (2014). Inhibitory effects of *Cnidium monnieri* fruit extract on pulmonary inflammation in mice induced by cigarette smoke condensate and lipopolysaccharide. Chin. J. Nat. Med..

[29] Jing C.L., Zhao J., Han X.B., Huang R.H., Cai D.S., Zhang C.S. (2018). Essential oil of *Syringa oblata* Lindl. as a potential biocontrol agent against tobacco brown spot caused by *Alternaria alternata*. Crop Prot..

[30] Bajpai V.K., Sharma A., Baek K.H. (2013). Antibacterial mode of action of *Cudrania tricuspidata* fruit essential oil, affecting membrane permeability and surface characteristics of food-borne pathogens. Food Control.

[31] Zhou H., Tao N., Jia L. (2014). Antifungal activity of citral, octanal and α-terpineol against *Geotrichum citri-aurantii*. Food Control.

